# Lung ultrasound assessment of pulmonary effects of large patent ductus arteriosus in extremely preterm infants beyond the transitional period

**DOI:** 10.1186/s13089-025-00450-4

**Published:** 2025-10-06

**Authors:** Thanaa Elhanafy, Nehad Nasef, Jenna Ibrahim, Rana Awadalla, Amish Jain, Adel Mohamed

**Affiliations:** 1 Department of Paediatrics, University of Toronto, Toronto, ON Canada; 2 Department of Paediatrics, Mount Sinai Hospital, 600 University Avenue, Toronto, ON M5G 1X5 Canada; 3 Department of Paediatrics, Mansoura University, Mansoura, Egypt

**Keywords:** Neonates, Lung ultrasound, Echocardiography, Patent ductus arteriosus, Preterm infant

## Abstract

**Background:**

Several studies have suggested a positive association between elevated lung ultrasound scores (LUS) and large patent ductus arteriosus (L-PDA), although findings remain inconsistent. Lung ultrasound score, a semi-quantitative measure of pulmonary aeration loss, has been proposed as a surrogate marker of excessive lung fluid, which may reflect the hemodynamic burden of a significant PDA. The aim of this study was to assess the association between LUS and L-PDA in preterm neonates beyond the initial transitional period and examine its correlations with echocardiographic measures of ductal shunting. This is a cohort retrospective study that included preterm infants born at < 29 weeks’ gestation who underwent LUS within 24 h of targeted neonatal echocardiography. Infants were categorized as having L-PDA (diameter ≥ 1.5 mm, left-to-right shunt) or no/small PDA (< 1.5 mm). Clinical characteristics, LUS, and echocardiographic parameters including PDA diameter, left atrial-to-aortic root (LA: Ao) ratio, and left ventricular output (LVO) were compared. Statistical analyses included univariate, multivariate, and correlation assessments.

**Results:**

Among 119 infants included in the analysis, 56 (47%) had L-PDA, and 63 (53%) had no or small PDA. Infants with L-PDA had significantly lower gestational age and higher rates of invasive ventilation. LUS, LA: Ao ratio, and LVO were significantly elevated in the L-PDA group (all *p* < 0.001). LUS correlated with PDA diameter (*r* = 0.27, *p* = 0.003) and respiratory severity score (*r* = 0.49, *p* < 0.001). Furthermore, LUS was found to be independently predictive for L-PDA (adjusted OR 1.5; 95% CI: 1.1–1.9). Each 1-point increase in LUS was associated with a 0.14 mm increase in PDA diameter. Inter-rater reliability for LUS was strong (IRR = 0.86).

**Conclusion:**

Beyond the transitional period, LUS was significantly associated with PDA size and independently predicted L-PDA in extremely preterm infants.

## Background

Patent ductus arteriosus (PDA) is one of the most common cardiovascular conditions in preterm infants, with a reported prevalence exceeding 60% among those born before 28 weeks’ gestation [1]. A persistent left-to-right shunt through the ductus arteriosus can result in pulmonary overcirculation, interstitial edema, and increased ventilator dependency [2, 3], thereby contributing to the development of bronchopulmonary dysplasia (BPD) [4, 5]. Despite decades of research, the diagnosis and management of PDA in preterm neonates remains a topic of ongoing debate in modern neonatology.

Echocardiography remains the gold standard for the diagnosis of PDA, offering both direct assessments of ductal diameter and flow pattern, and indirect evaluations of shunt volume and its systemic effects [6]. Given the complexity of defining hemodynamically significant PDA (hsPDA), a pragmatic classification has been used categorizing PDAs as large (L-PDA; defined as a diameter ≥ 1.5 mm with predominantly left-to-right flow) versus absent or small (diameter < 1.5 mm) [7]. Studies have shown that echocardiographic signs of hsPDA often precede overt clinical manifestations by nearly two days, highlighting the need for complementary bedside tools to enhance early detection [8].

Lung ultrasound (LU) has emerged as a promising, non-invasive modality for assessing pulmonary congestion in neonates, with potential utility in estimating the pulmonary impact of PDA. Several studies [9–12] have reported a positive correlation between elevated LU scores (LUS) and hsPDA. However, evidence from the early transitional period (first 72 h of life) remains inconsistent, as Mohamed et al. reported no significant association between LUS and PDA during this time, likely due to confounding effects of early parenchymal lung disease [7].

 We hypothesize that the association between LUS and PDA becomes more evident beyond the transitional phase, when acute respiratory pathology has subsided and the hemodynamic impact of a persistent PDA is more pronounced. In this study, we evaluated the relationship between LUS and PDA in preterm infants who underwent both lung ultrasound and echocardiography between days 7 and 14 of life. By targeting this timeframe, the study aims to clarify the diagnostic utility of LUS in identifying clinically significant PDA and to support its role as a non-invasive adjunct to echocardiography in the ongoing assessment of PDA beyond the early neonatal period.

## Methods

### Study design, setting, and population

This retrospective cohort study was conducted at a Canadian tertiary-level neonatal intensive care unit (NICU) between January 2020 and December 2024. Eligible participants included preterm infants born at < 29 weeks’ gestation who underwent both clinical echocardiography and LU within a 24-hour interval between days 7 and 14 of life, with echocardiographic documentation of PDA characteristics. Infants were excluded if they had chromosomal abnormalities, major congenital malformations, or if only one of the two imaging modalities (echocardiography or LU) was performed during the study period. The study received approval from the institutional research ethics board, and all procedures were conducted in accordance with the principles outlined in the Declaration of Helsinki.

### Lung ultrasound assessment

Lung ultrasound (LU) examinations were performed using a portable ultrasound system (Z.One PRO, Mindray North America) equipped with a high-frequency linear transducer (L20-5). To minimize overestimation of lung ultrasound scores (LUS) due to gravity-dependent pulmonary changes, infants were placed in the supine position for at least one hour prior to scanning [13]. Each lung was systematically assessed across three regions: upper anterior, lower anterior, and lateral zones. All scans were conducted by trained personnel with demonstrated expertise in neonatal LU. Acquired images were securely archived on a password-protected external hard drive. Two independent, experienced reviewers blinded to the infants’ clinical status evaluated the LU images and assigned scores using a standardized 18-point scoring system [14]. This semi-quantitative method grades each of six chest regions (three per hemithorax) from 0 to 3 based on lung aeration patterns: 0 = normal aeration (A-lines or ≤ 2 isolated B-lines); 1 = multiple, non-coalescent B-lines; 2 = coalescent B-lines producing a white lung appearance without consolidation; and 3 = consolidation with tissue-like pattern ± air bronchograms. Higher total scores indicate greater loss of aeration and correlate with clinical and radiographic markers of pulmonary disease severity.

### Targeted neonatal echocardiography assessment

Our unit operates a well-established targeted neonatal echocardiography (TNE) consultation program. Within this framework, TNEs are conducted either by experienced TNE-certified staff physicians or by trained sonographers and fellows under direct supervision. Throughout the study period, all echocardiographic assessments were performed using a GE E95 ultrasound system equipped with a 12 MHz phased-array transducer, using our units previously published standardized TNE imaging protocol [15]. TNE studies are interpreted and formally reported on the same day by one of four credentialed TNE staff physicians. As part of routine clinical documentation, all TNE reports include an assessment of PDA status, along with key hemodynamic indicators reflective of ductal shunt volume. For the purpose of this study, the following parameters were extracted from each TNE report: presence of PDA, PDA diameter (in mm), left ventricular output (LVO), and the left atrial-to-aortic root diameter ratio (LA: Ao), measured according to standardized methods [16].

All infants underwent echocardiography to assess the presence and severity of PDA (categorized as hemodynamically significant [HsPDA], small/ no PDA). Only infants with HsPDA received medical treatment, which consisted of ibuprofen (20 mg/kg IV or PO initially, followed by 10 mg/kg daily for two additional doses, for a total of three doses). Lung ultrasound (LUS) was performed within 24 h of the initial echocardiogram. A follow-up echocardiogram was typically performed 72 h later, after completion of the treatment course.

### Study cohort grouping

The definition of hemodynamically significant PDA (hsPDA) remains a topic of debate, with no universally accepted criteria [17, 18]. Although several hsPDA scoring systems have been proposed, they have not been validated [19, 20]. For the purposes of this study, we applied a pragmatic classification to stratify the cohort into two groups: infants with large PDA (L-PDA), defined as a ductal diameter ≥ 1.5 mm with predominantly left-to-right unrestrictive shunting, and those with absent or small PDA, defined as a ductal diameter < 1.5 mm.

### Clinical data collection

Clinical data were extracted from the patients’ electronic medical records. The dataset included a range of demographic and clinical variables: gestational age (GA), sex, birth weight (BW), mode of delivery, and antenatal steroid exposure. Additional parameters included surfactant administration, invasive mechanical ventilation (IMV) use, development of BPD, and mortality prior to hospital discharge.

Respiratory variables recorded at the time of lung ultrasound included mode of ventilation, mean airway pressure (MAP), fraction of inspired oxygen (FiO₂), and the calculated respiratory severity score (RSS = FiO₂ × MAP). Lung ultrasound scores and TNE findings related to PDA, specifically PDA diameter, LA: Ao, and LVO were also collected.

### Outcomes

The primary aim of this study was to investigate the association between lung ultrasound scores (LUS) and the presence of a L-PDA, as determined by TNE during the second week of life. The secondary objective was to examine the correlation between LUS and key echocardiographic parameters, including PDA diameter, LA: Ao, and LVO.

### Data analysis

Statistical analysis was performed using SPSS statistical software (version 23; IBM Corporation, Armonk, NY, USA). For continuous parametric variables, the Student’s t-test was used to compare means between two groups. For continuous non-parametric variables, the Mann-Whitney U test was used to compare medians between two groups. For categorical variables, the Chi-square test or Fisher exact test was used depending on the number of observations in each category. The Kolmogrov Smirnov test was used to examine the distribution of data. Pearson’s correlation coefficient test was used to correlate between LUS with RSS score, LUS with PDA diameter, LUS with LA: Ao ratio, LUS with LVO, and RSS score with PDA diameter. A binary logistic regression adjusted for GA, IMV, RSS, and antenatal steroid was performed to detect independent predictability of LUS for large PDA. Hosmer and Lemshow test was used to detect goodness of fit for each of the regression models. A p-value of less than 0.05 will be considered statistically significant. The data are reported as mean ± standard deviation, median (inter-quartile range), or number (percentage) depending on the variable type. Interclass correlation coefficient was calculated to assess the reproducibility of the scan on repeated exams performed by the two examiners on 30 infants.

## Results

Of the 119 preterm infants included in the study, 56 had a large PDA (L-PDA), and 63 had no or small PDA. The L-PDA group had significantly lower median GA (24.7 weeks [IQR: 24.0–26.0] vs. 25.7 weeks [IQR: 24.7–27.0], *p* = 0.002) and lower birth weight (645 g [IQR: 565–817] vs. 740 g [IQR: 610–890], *p* = 0.026). A higher proportion of infants with L-PDA required invasive mechanical ventilation, but it didn’t reach statistical significance (59% vs. 41%, *p* = 0.054). Mean airway pressure, FiO₂, and respiratory severity score (RSS) did not differ significantly between groups (Table 1).

**Table 1:** Characteristics of study participants

	L-PDA (*n* = 56)	No/small PDA (*n* = 63)	*P* value
GA at birth (wk), median (IQR)	24.7 (24.0–26.0)	25.7 (24.7–27.0)	0.002
Birth weight (g), median (IQR)	645 (565–817)	740 (610–890)	0.026
Male, n (%)	29 (52%)	32 (51%)	0.91
SGA, n (%)	2 (4%)	4 (6%)	0.49
Antenatal steroids, n (%)	49 (87%)	48 (76%)	0.17
Caesarean delivery, n (%)	24 (43%)	43 (86%)	0.005
Single dose Surfactant, n (%)	19 (34%)	32 (51%)	0.10
Multiple doses of Surfactant, n (%)	31 (55%)	25 (40%)	0.15
Failed extubation, n (%)	12 (21%)	13 (21%)	1.0
BPD (moderate, severe), n %	47 (84%)	50 (79%)	0.61
Mortality prior to discharge, n %	6 (11%)	3 (5%)	0.22
Respiratory Support at the Time of Assessment			
IMV at the scan, n %	33 (59%)	26 (41%)	0.054
MAP at the scan median (IQR)	10 (8–12)	9 (7–11)	0.47
FiO2 at the scan, median (IQR)	0.3 (0.23–0.37)	0.27 (0.23–0.33)	0.19
RSS at scan, median (IQR)	3.2 (1.9–4.2)	2.6 (1.9–3.5)	0.18

L-PDA: Large- patent ductus arteriosus; GA: gestational age; IQR: interquartile range; SGA: small for GA; SNAPPE-II: Score for Neonatal Acute Physiology with Perinatal Extension-II; BPD: bronchopulmonary dysplasia; IMV: Invasive Mechanical Ventilation; NIV: Non-invasive ventilation; MAP: Main Airway Pressure; FiO2: fraction of inspired oxygen: RSS: Respiratory severity score (= FiO2 x MAP); SD: standard deviation

Notes: the reported p-values were based on the comparison between two groups using Chi-square test or Fisher’s exact test* for categorical variables and Student t test or Wilcoxon-Rank-Sum test as appropriate for continuous variables

**Fig. 1:**
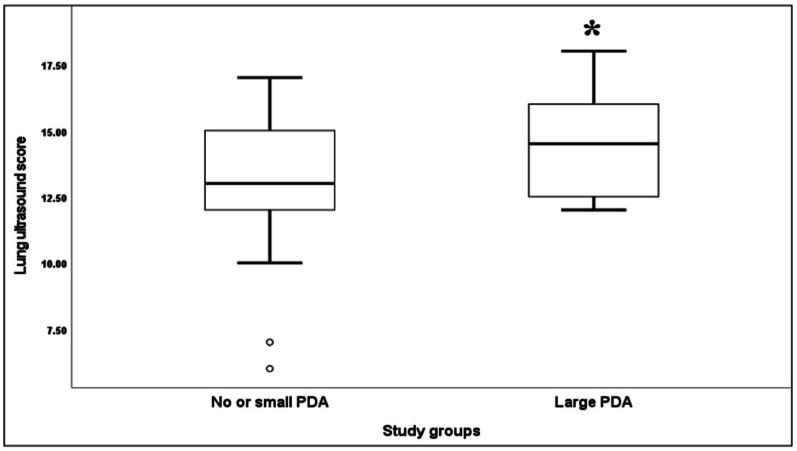
Comparison of LUS Between two groups

**Fig. 2:**
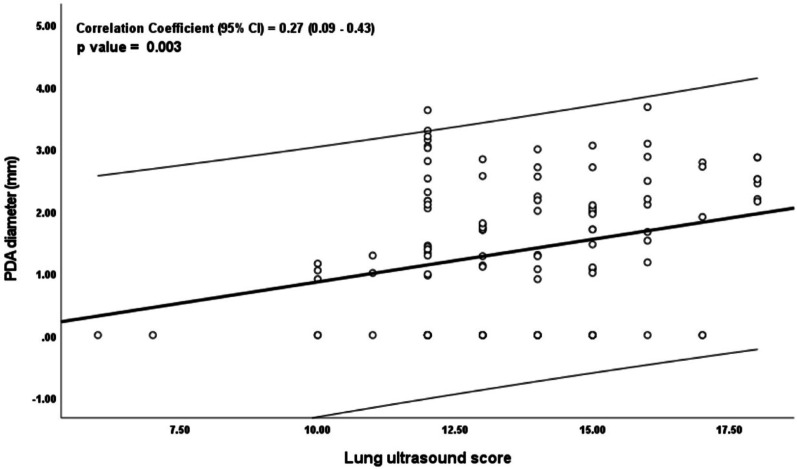
Correlation between PDA Diameter (mm) and LUS

**Fig. 3:**
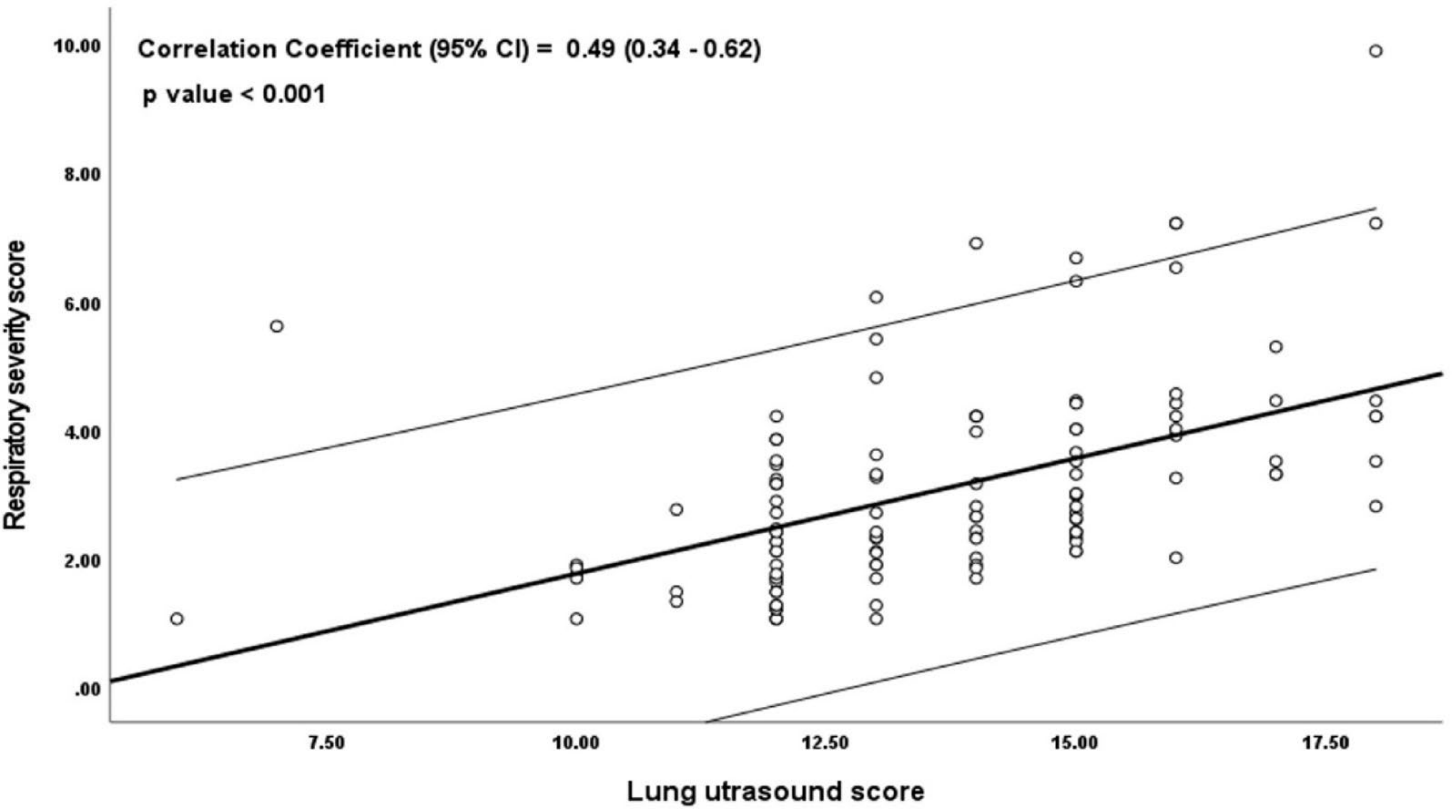
Correlation between RSS and LUSS for the study Cohort

**Fig. 4:**
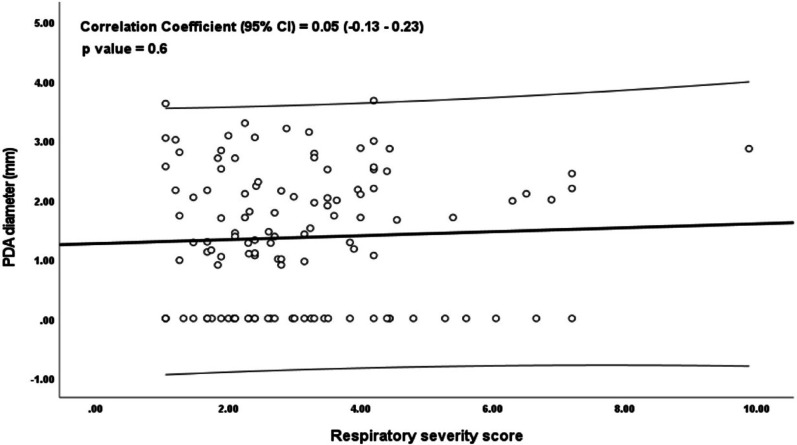
Correlation between PDA Diameter (mm) and RSS

Lung ultrasound scores (LUS) were significantly higher in the L-PDA group (*p* < 0.001), as were echocardiographic indices: LVO and LA: Ao ratio ( *p* < 0.001, and 0.003 respectively) (Table 2; Fig. 1). LUS correlated significantly with PDA diameter (*r* = 0.27, *p* = 0.003), LA: Ao ratio (*r* = 0.23, *p* = 0.046, and RSS (*r* = 0.49, *p* < 0.001), while no correlation was observed between LUS and LVO (*r* = 0.18, *p* = 0.06) or between PDA diameter and RSS (*r* = 0.04, *p* = 0.70) (Figs. 2, 3 and 4).

**Table 2:** Comparison of LUS and echocardiographic indices between groups

	L-PDA *n* = 56	No/small PDA *n* = 63	*P* value
LUS, median (IQR)	14.5 (12–16)	13 (12–15)	< 0.001
La: Ao, median (IQR)	1.8 ± 0.4	1.5 ± 0.4	0.003
LVO (ml/kg/min), mean (SD)	332.4 ± 85	246.9 ± 64	< 0.001

LUS: Lung ultrasound score; PDA: Patent Ductus Arteriosus; La: Ao: left atrial-to-aortic root ratio; LVO: left ventricular output

The reported p-values were based on the comparison between two groups Wilcoxon-Rank-Sum test

In logistic regression analyses, LUS was independently associated with the presence of L-PDA (unadjusted OR 1.4; 95% CI: 1.2–1.8; adjusted aOR 1.5; 95% CI: 1.1–1.9) (Table 3). Linear regression showed each 1-point increase in LUS was associated with a 0.14 mm increase in PDA diameter (95% CI: 0.04–0.25, *p* = 0.01) (Table 4). Inter-rater agreement for LUS was high (ICC = 0.86; 95% CI: 0.73–0.93, *p* < 0.001).

**Table 3:** Multivariate binary logistic regression analyses

	Odds Ratios (95% CI) L-PDA vs. no-PDA/small PDA			
	Unadjusted (95% CI)	p value	Adjusted (95% CI)	p value
LUS (one unit increase in score)	1.4 (1.2–1.8)	< 0.001	1.5 (1.1–1.9)	0.003
RSS (one unit increase in score)	1.2 (0.9–1.5)	0.12	0.8 (0.6–1.1)	0.15

CI: Confidence Interval

Adjusted OR was based on the multiple logistic regression model adjusted for LUS, GA, IMV, RSS, and antenatal steroid

**Table 4:** Multivariate linear regression analyses

	Beta Coefficients (95% CI) PDA size in mm	
	Beta (95% CI)	p value
LUS (one unit increase in score)	0.14 (0.04–0.25)	0.01
GA (One week increase in GA)	-0.10 (− 0.24–0.04)	0.15
RSS (one unit increase in score)	-0.11 (-0.26–0,04)	0.14

“In a linear regression analysis, each unit increase in LUS was associated with 0.14 (95% CI 0.04–0.25, *p* = 0.01) mm increase in PDA diameter

## Discussion

In this cohort study of preterm infants born at < 29 weeks’ gestation, we found that LUS was significantly associated with both the presence and size of PDA during the second postnatal week. Infants with large PDAs (L-PDA) had significantly higher LUS compared to those with no or small PDA. Importantly, LUS remained an independent predictor of L-PDA even after adjusting for key clinical variables including GA, IMV, RSS, and antenatal steroid exposure. Additionally, linear regression analysis demonstrated a positive correlation between LUS and PDA diameter, supporting the role of LUS as a surrogate marker of the pulmonary hemodynamic burden imposed by a persistently patent ductus.

This observed association between LUS and L-PDA is both biologically plausible and clinically relevant. A large PDA facilitates left-to-right shunting, resulting in pulmonary overcirculation, interstitial fluid accumulation, and impaired alveolar gas exchange. These pathophysiological changes are reflected on lung ultrasound as increased B-lines, pleural line irregularities, and subpleural consolidations. Consequently, the LUS increases, serving as a non-invasive marker of the pulmonary sequelae of a hemodynamically significant PDA (hsPDA) [21, 22].

By conducting our assessments beyond the immediate transitional period (i.e., after day 7), our study minimizes the confounding impact of RDS, which commonly elevates LUS in the early postnatal days [14]. This timing allows for a more accurate attribution of elevated LUS to PDA-related pulmonary congestion rather than primary surfactant deficiency.

Our findings are in agreement with prior studies suggesting that elevated LUS is indicative of increased pulmonary fluid burden secondary to significant left-to-right shunting [9–12]. The higher LUS observed in the L-PDA group likely reflects pulmonary edema resulting from increased pulmonary blood flow. While some studies have reported inconsistent associations, particularly when LUS is performed within the first 72 h of life [7], this study’s focus on the second postnatal week allowed us to better isolate the contribution of PDA-related hemodynamic changes to LUS.

Furthermore, we observed a strong positive correlation between LUS and both PDA diameter and RSS, suggesting that LUS captures not only the anatomical dimension of pulmonary involvement, reflected by ductal size, but also the functional impact on respiratory status. In contrast, no correlation was found between PDA diameter and RSS. While this may initially appear unexpected, it is clinically and physiologically plausible. PDA diameter represents a static anatomical measure and does not consistently reflect the hemodynamic significance or the extent of respiratory compromise, which are influenced by multiple factors including pulmonary vascular resistance, myocardial function, and overall lung maturity [19, 23].

LU, on the other hand, directly visualizes the pulmonary consequences of shunting, such as interstitial edema and alveolar fluid that may not be predicted by PDA size alone. Therefore, the significant correlations between LUS and both PDA diameter and RSS highlight its ability to integrate structural and physiological information, whereas PDA diameter alone remains a static anatomical parameter with limited clinical predictive value [24, 25].

Additionally, our study confirmed the reproducibility of LUS scoring in this population, as reflected by the excellent inter-rater reliability (ICC = 0.86). This is consistent with previous studies that have validated LUS as a reliable and operator-sensitive bedside tool when performed by trained clinicians [26].

This study has several strengths, including a relatively large sample size, standardized imaging protocols for both lung and cardiac ultrasound, and adjustment for multiple potential confounders through multivariate analysis. However, certain limitations should be acknowledged. The retrospective, single-center design may limit the generalizability of our findings and introduces the potential for information bias. Additionally, although inter-rater agreement was high, LUS interpretation remains operator-dependent. Finally, while LUS correlates with PDA size and associated pulmonary effects, it does not replace echocardiography for comprehensive assessment of PDA hemodynamics, such as flow pattern or left heart volume load.

## Conclusion

Our findings support the utility of lung ultrasound as a non-invasive, bedside modality that reflects the pulmonary impact of large PDA in preterm infants beyond the transitional period. LUS may serve as a valuable adjunct to echocardiography for identifying infants at risk of PDA-related pulmonary compromise and can potentially aid in risk stratification and individualized clinical decision-making.

## Data Availability

The datasets used and/or analyzed during the current study are available from the corresponding author on reasonable request.

## Abbreviations

LU: Lung ultrasound
LUS: Lung ultrasound score
TNE: Targeted Neonatal Echocardiography
PDA: Patent ductus arteriosus
L-PDA: Large Patent Ductus Arteriosus
hsPDA: Hemodynamically significant PDA
LA:Ao: Left Atrial-to-Aortic root Ratio
LVO: Left Ventricular Output
RSS: Respiratory Severity Score
SD: Standard deviation
